# Items analysis of the Frailty Index (FI-35): Insight in the contribution of each item to the level of frailty

**DOI:** 10.1371/journal.pone.0258588

**Published:** 2021-11-08

**Authors:** Xiaohong Zhang, C. P. Van Der Schans, Yanhui Liu, W. Krijnen, J. S. M. Hobbelen

**Affiliations:** 1 Tianjin University of Traditional Chinese Medicine, Tianjin, China; 2 Hanze University of Applied Sciences, Research Group Healthy Ageing, Allied Health Care and Nursing, Groningen, The Netherlands; 3 Department of Rehabilitation Medicine, University of Groningen, University Medical Center Groningen, Groningen, The Netherlands; 4 Department of Health Psychology, University of Groningen, University Medical Center Groningen, Groningen, The Netherlands; 5 Department of General Practice and Elderly Care Medicine, University of Groningen, University Medical Center Groningen, Groningen, The Netherlands; IRCCS E. Medea, ITALY

## Abstract

**Background:**

The FI-35 is a valid multidimensional Chinese frailty assessment instrument. Like other scales, functional measures rely on the information the total score provides. Our research aimed to analyze the contribution of each item.

**Methods:**

Descriptive statistics were used to summarize the sample characteristics. The expected item score (EIS) was used to determine how the items contribute to the generic measure of frailty.

**Results:**

This study showed that most of the EIS curves increased across the entire range of frailty levels, and most of the items discriminate relatively well over the entire frailty range. Items differentially contributed to the total frailty score and differentially discriminated between frailty levels.

**Conclusions:**

Although nearly all items monotonically increased with frailty levels, there were large differences between items in their ability to differentiate between persons being either weakly, moderately or highly frail.

## Background

The World Health Organization (WHO) reported that the proportion of the total population of the world over 60 years old will nearly double to 22% from 2015 to 2050 [1]. In China, the population aged above 60 will rise 24.6% by 2050 [2]. The Global strategy and action plan on ageing and health pointed out that to develop a healthy aging society, physical and mental capacities and relevant environmental factors should be considered [3]. However, the current metrics are limited, which prevents a complete understanding of health issues and the usefulness of interventions in the field of aging. Thus, focused research is essential to promoting healthy aging.

This rapid increase in aging brings a variety of problems and challenges to the healthcare system, one of the most important of which is the development of frailty in older people. Frailty is an age-related condition commonly defined as a multidimensional geriatric syndrome with decreased reserves of energy, physical abilities, cognition, and adaptive abilities, which give rise to vulnerability [4,5]. Frailty increases the likelihood of falls, fractures, iatrogenic complications, early mortality and other adverse health events, and frailty can occur in the absence of comorbidities [6]. The number of older adults with frailty has been increasing because of the rapid expansion of the aging population, and it is necessary for individualized care and interventions to focus on the diversity and heterogeneity within the aged population.

It is common to use frailty measurements for the purpose of prognosis in medical specialties. Frailty has been quantified using different instruments: the frailty phenotype (FP) [7], the frailty index (FI) [8], the Tilburg frailty indicator (TFI) [9], the Groningen frailty index (GFI) [10] and the frailty index scale (FI-35) [11]. The FP assesses frailty based on five criteria (weight loss, exhaustion, weakness, slow walking speed, and low physical activity) based on patients’ poor functional performance [7]. In contrast, the FI recognizes the influence of other nonphysical factors and measures frailty by counting the number of health-related physical and psychological deficits accumulated by a person [8]. With the development of the TFI and GFI, the multidimensionality of frailty was further expanded to include a social domain [9,10]. In addition to the physical, psychological and social domains included in the FP, FI, GFI and TFI, the Chinese frailty index FI-35, consisting of 35 items, also incorporates an assessment of environmental factors that may influence frailty status [11].

Regarding the conceptualization of scoring frailty, frailty in previous research has been measured based only on traditional approaches of using a cumulative score or, in the best case, total scores for frailty in specific domains. However, total scores provide information concerning only the level of frailty and are limited with regard to the content of frailty, and total scores provide no direction regarding the choice of an intervention to reduce frailty, which is the focus of a clinician. Most of the studies have ignored the individual items that might provide important information, such as their contribution to the total frailty score and differences between levels of frailty. Similarly, it is currently unknown to what extent a pattern of answers across items may result in high or low certainty with respect to the actual level of the person’s frailty. Furthermore, specific patterns of answers can widely differ between persons in the sense of being rather broad and spread across all domains or being rather concentrated in a few specific domains or on specific items in specific domains. It is important that multidimensionality as well as different patterns of frailty are recognized in the clinical setting to improve care for older persons.

Item response theory (IRT) is a powerful tool for supplying detailed information about item functioning and it can be used efficiently to find the dimensions of an instrument [12]. IRT is used in scale development and modification to assess the health, like anxiety and sleep quality [13,14]. Nowadays, many kinds of different IRT models have been proposed. Models can be classified into parametric (PIRT) and non-parametric IRT models (NIRT) [15]. Although IRT are generally used, nonparametric models and methods are becoming more popular [16]. Compared with more commonly used IRT, the main advantage of NIRT is that they relax some of the strong assumptions about the nonlinear behaviour of response probabilities which are invoked by IRT models [17]. NIRT specifying the underlying latent traits interact with the item’s characteristics on the ordinal scale [18]. This method was selected because its Expected Item Scores (EIS) provide a detailed insight into how each item performs in relation to the underlying concepts of the frailty. The purpose of this study is to determine the contribution of individual items to frailty and the degree to which this contribution depends on the level of frailty.

## Methods

### Setting and participants

We used data from a cross-sectional study that was conducted in the city of Tianjin, China. This survey collected data from November 2017 to February 2018 using the FI-35 scale among older adults aged 60 years and over living in a community who had the ability to fill in the questionnaire. Furthermore, the selection criteria for the sample of participants were being able to communicate, being conscious, and being able to voluntarily participate in the study.

### Ethical procedures

Permission for the study was granted by Tianjin University of Traditional Chinese Medicine. All procedures performed in studies involving human participants were in accordance with the ethical standards of Tianjin University of Traditional Chinese Medicine. Participants were informed prior to their participation about the purpose of study and the consequences of the provided information, and they signed informed consent.

### Measures

The FI-35 consists of 35 self-reported items that are organized into 4 domains (physical, psychological, social and environmental). These four domains each have several subdomains, consisting of 3 or 4 items. The physical domain has five subdomains: nutrition (3 items), exercise (4 items), muscle strength (3 items), vigor (3 items), and sleep quality (3 items). The psychological domain has two subdomains: emotion (3 items) and cognition (4 items). The social domain has two subdomains: role (3 items) and social contact (3 items). The environment domain also has two subdomains: environment (3 items) and adaptability (3 items) [11]. All participants were asked to rate each of the 35 items of the FI-35 as “no”, “sometimes” and “yes”, which were scored as 0, 0.5, and 1, respectively. Higher scores are intended to indicate higher levels of frailty.

### Statistical analyses

Descriptive statistics were used to summarize the sample characteristics with IBM SPSS Statistics 21.0. To allow a more in-depth analysis of item properties, we performed non-parametric item response analysis of the FI-35 data using R statistical software version 3.1.3 (R Foundation for Statistical Computing), and specifically, we used the functionality of kernel smoothing in item response theory (KSIRT) [19].

First, to determine the associations between each item, which consisted of the ordered categorical scores 0, 0.5, or 1 based on the responses (i.e., not, sometimes and fully endorsing the statement) and the continuous latent frailty trait, the polyserial correlation coefficient was computed for each of the items.

Second, to determine how individual items contribute to the generic measure of frailty, a flexible non-parametric approach of KSIRT was employed. This provides an analysis for each item in a specific EIS figure that shows how the expected item score (EIS), which ranges from zero and one, depends on the expected total frailty score. Vertical dashed lines represent the 5th, 25th, 50th, 75th and 95th percentiles of the expected scale score, and the (nearly always) ascending curves indicate that older people with higher frailty levels were increasingly likely to fully endorse an item [19]. These EIS plots indicate whether and to what degree the probability of endorsing an item increases monotonically with increasing levels (across subjects) of the underlying frailty trait. In this way, these EIS plots show how the FI-35 items differentiate among nonfrail, moderately frail and highly frail persons.

## Results

### Study sample characteristics

**Fig 1:**
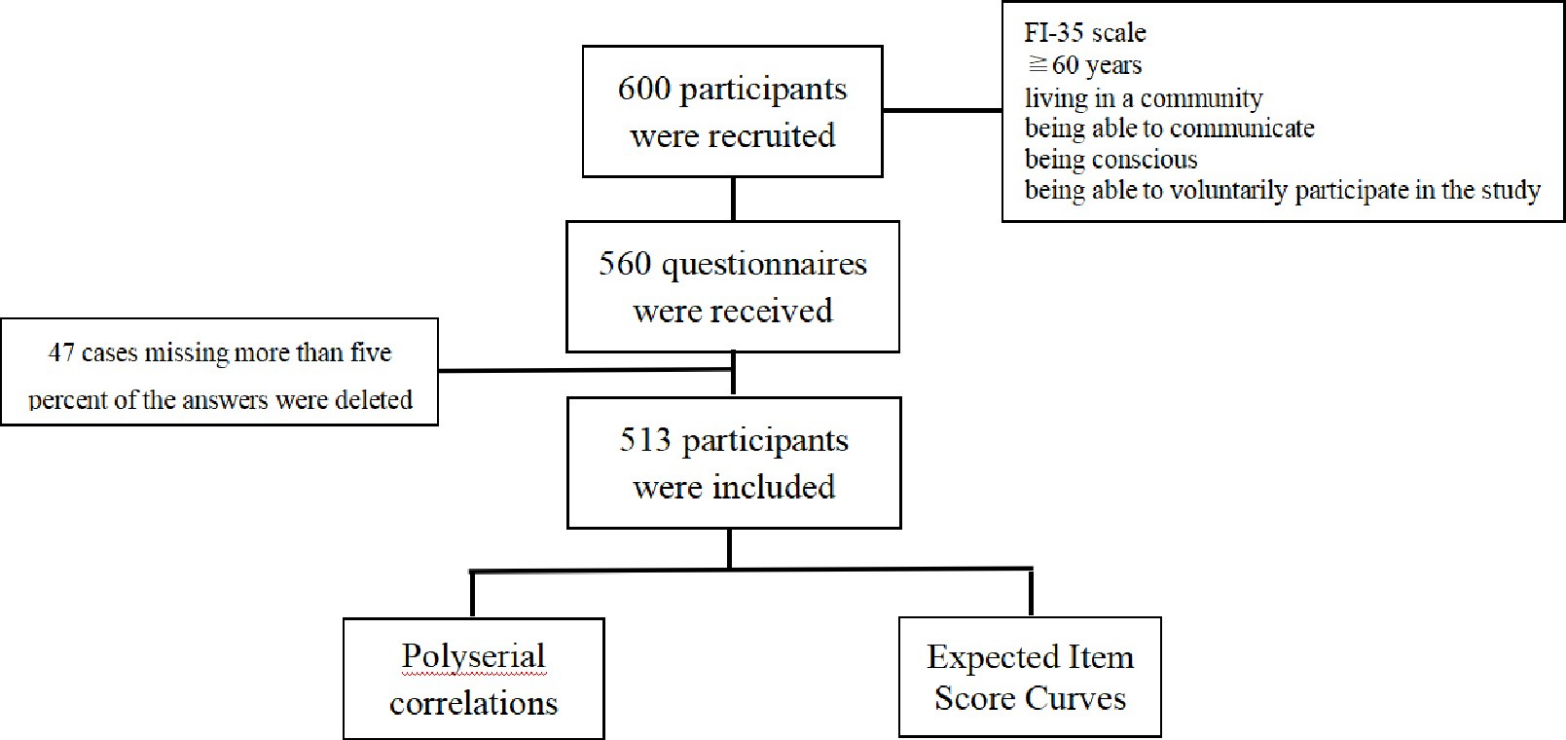
Flowchart of the research.

A total of 600 participants were recruited from three older adult communities in Tianjin. From these, 560 questionnaires were received, and we deleted cases missing more than five percent of the answers. We included 513 participants in the present analyses, corresponding to an effective response rate of 91.6%. The planned flow flowchart of the research is presented in Fig 1. The respondents were predominantly female (285, 55.6%) and married (429, 83.6%) with a mean (SD) age of 69.6 (7.2) years, the age ranges from 60 to 91. The FI-35 score ranged from 0.00 to 0.89, with a median of 0.31. Using a cut-off score of FI ≥ 0.23 for frailty, 67.6% of individuals were considered frail.

A total of 202 (39.4%) participants had an education level of college degree or above. The largest number of people were in the middle-income group of “2000–3999 Yuan” (223, 43.47%). Other main sociodemographic characteristics of the study sample are presented in Table 1.

**Table 1:** Characteristics of the study sample (n = 513).

Variable	Group	Number	Percentage (%)
Gender	Male	228	44.4
	Female	285	55.6
Age	60~64	134	26.1
	65~69	182	35.5
	70~74	65	12.7
	75~79	93	18.1
	>80	39	7.6
Marriage	Married	429	83.6
	Unmarried	3	0.6
	Divorced	30	5.9
	Widowed	51	9.9
Children	No	17	3.3
	Yes	496	96.7
Education level	Never attended school	48	9.4
	Primary school	47	9.2
	Junior school	134	26.1
	High school	82	15.9
	College degree or above	202	39.4
Income (yuan/month)	<1000	42	8.2
	1000–1999	53	10.3
	2000–3999	223	43.5
	≥4000	190	37.0
Smoking	Never smoked	382	74.5
	Gave up smoking	74	14.4
	Smoking	57	11.1
Drinking	Never drank	380	74.1
	Gave up drinking	90	17.5
	Drinking	43	8.4
Exercise	Never	160	31.2
	1–2 times/week	59	11.5
	3–4 times/week	94	18.3
	Every day	200	39.0
Extensive hobbies	Yes	276	53.8
	No	237	46.2

### Item response data analysis: Polyserial correlations

The size of the polyserial correlation of each item with the latent frailty concept was positive, indicating an overall positive association of each item with generic frailty traits. That is, increases in generic frailty were associated with greater probabilities of endorsement of an item (Table 2).

**Table 2:** Descriptive data for the items of the FI-35.

Subdomain	Item	Proportions of persons endorsing answer categories			Polyserial correlation of each item
		0	0.5	1	
Nutrition	1. Your weight has declined more than 5 kg	0.84	—	0.16	0.39
	2. Your appetite has decreased	0.81	—	0.19	0.30
	3. Your eating habits has changed	0.82	0.01	0.17	0.37
Exercise	4. Your walking ability has changed	0.64	—	0.36	0.49
	5. You walked more slowly	0.83	—	0.17	0.64
	6. It’s harder than before to go upstairs (downstairs)	0.57	—	0.43	0.40
	7. You can do some energy-consuming activities	0.61	0.01	0.38	0.16
Muscle strength	8. Your grip strength is weaker than before	0.77	—	0.23	0.45
	9. You can lift 5–10 kg	0.77	—	0.23	0.49
	10. Getting up from the chair is harder than before	0.81	—	0.19	0.56
Vigor	11. It was harder for you to do anything than before	0.53	0.32	0.15	0.47
	12. You often feel tired	0.55	0.31	0.14	0.47
	13. You are always listless	0.63	0.23	0.14	0.50
Sleep	14. You’re getting less sleep	0.55	0.01	0.44	0.50
	15. Your sleep is more interrupted than before	0.51	—	0.49	0.48
	16. You need medication to help you sleep	0.53	—	0.47	0.55
Emotion	17. You are more likely to feel down than before	0.81	0.10	0.09	0.08
	18. You feel lonelier than before	0.82	0.15	0.03	0.31
	19. You are not as confident as before	0.81	0.14	0.05	0.23
Cognition	20. You become forgetful	0.73	—	0.27	0.45
	21. You can express your thoughts as clearly as before.	0.73	—	0.27	0.45
	22. Your ability to solve problems is as good as before	0.75	—	0.25	0.47
	23. It is harder to focus on one thing than before	0.27	0.28	0.45	0.05
Role	24. You can engage in multiple roles (caring for families)	0.81	—	0.19	0.37
	25. Your daily life needs assistance from others	0.89	0.03	0.08	0.32
	26. Your confidence is less than before when you deal with things	0.31	0.49	0.20	0.28
Social contact	27. You can get support from others	0.12	0.02	0.86	0.21
	28. You keep in touch with your family and friends	0.13	0.03	0.84	0.28
	29. You are willing to participate in social activities	0.09	0.03	0.88	0.21
Environment	30. You are worse at adapting to temperature changes	0.76	—	0.24	0.42
	31. You are more sensitive to the environment	0.81	—	0.19	0.45
	32. Your living condition is more convenient than before you live	0.65	—	0.35	0.43
Adaptability	33. You can get information you need (like news)	0.81	0.02	0.17	0.53
	34. You can use new facilities (like computer, community equipment)	0.68	0.18	0.13	0.34
	35. You can understand some new ideas	0.65	0.22	0.13	0.30

Response alternatives were yes (1) and no (0), with a response category in the middle which means sometimes (0.5); higher scores represent more frailty.

### Expected item score curves

**Fig 2:**
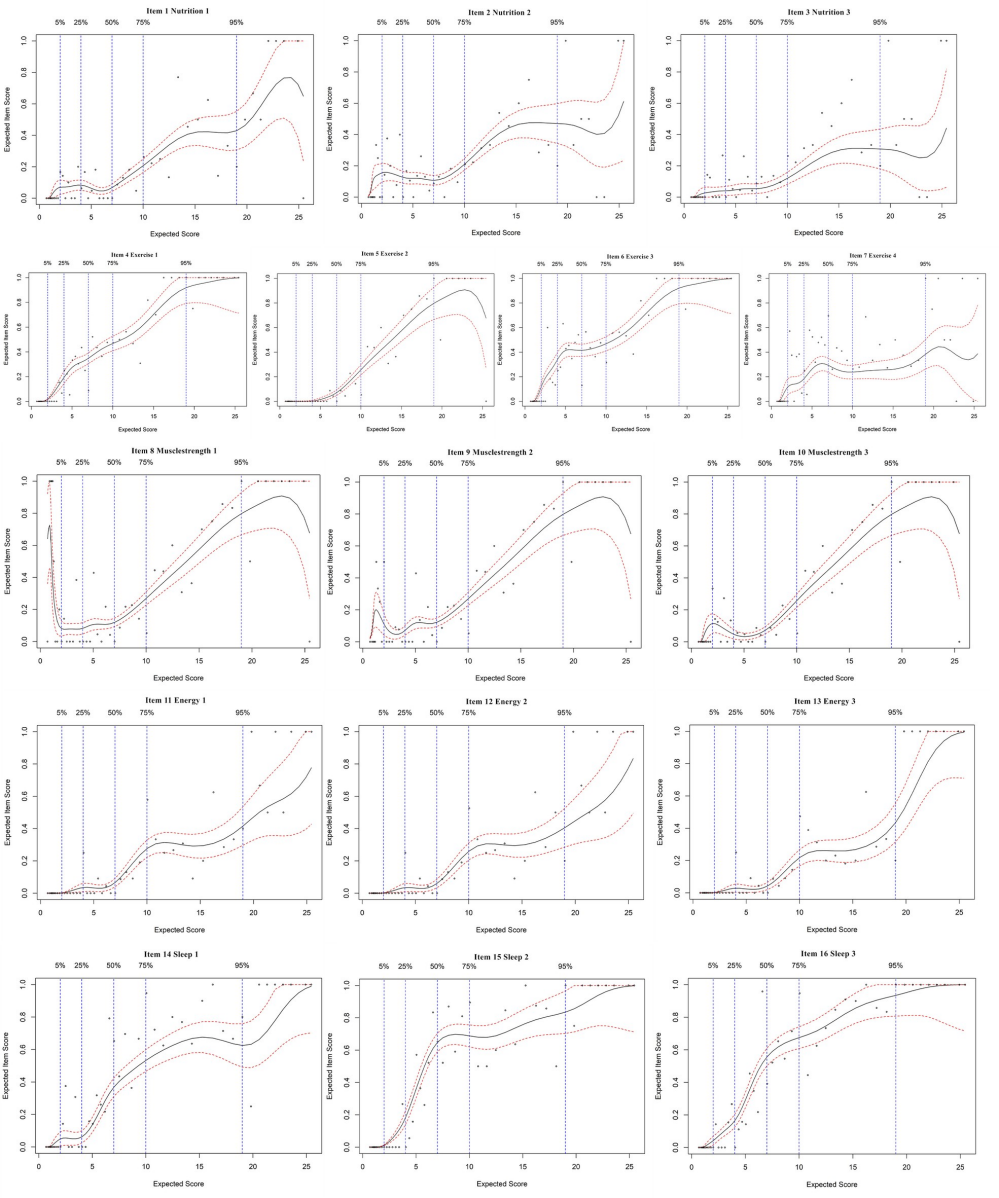
Expected item score curves for physical domain.

**Fig 3:**
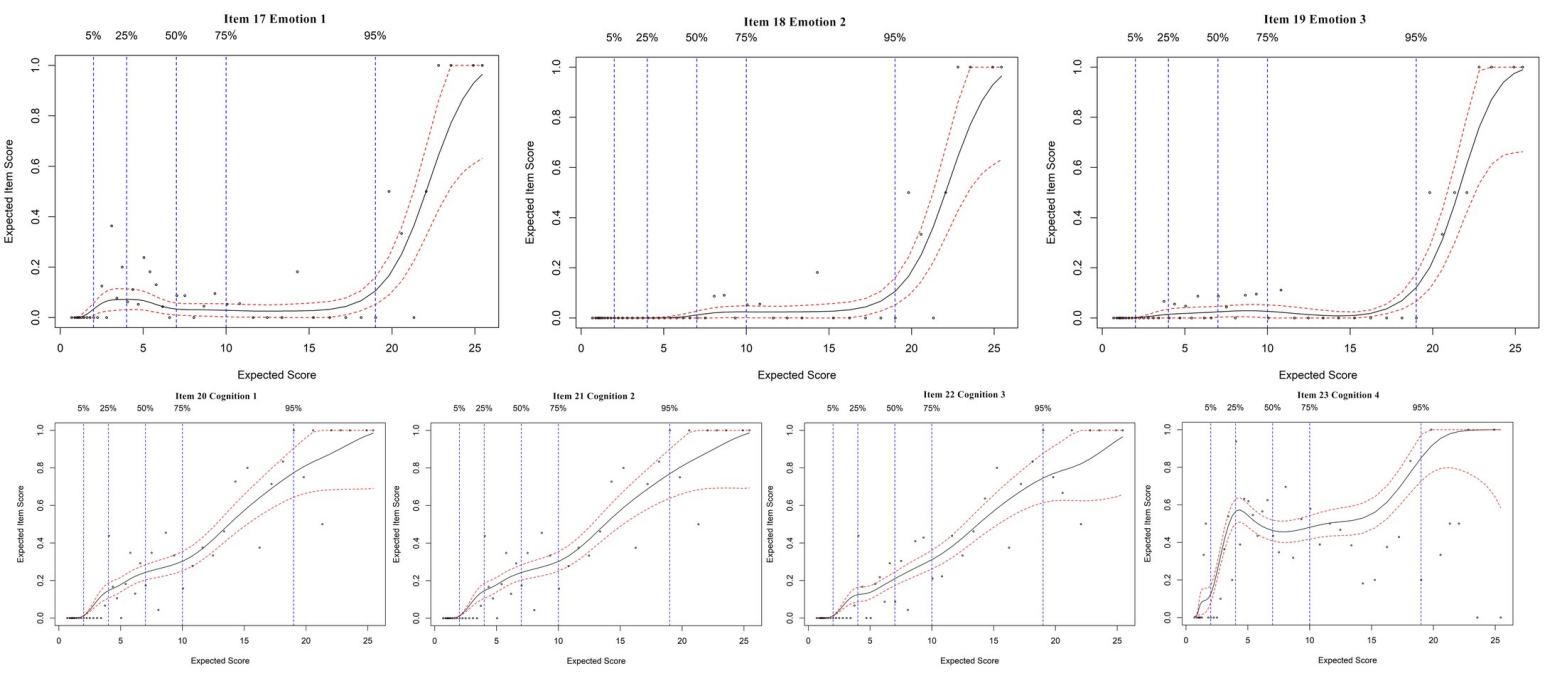
Expected item score curves for psychological domain.

**Fig 4:**
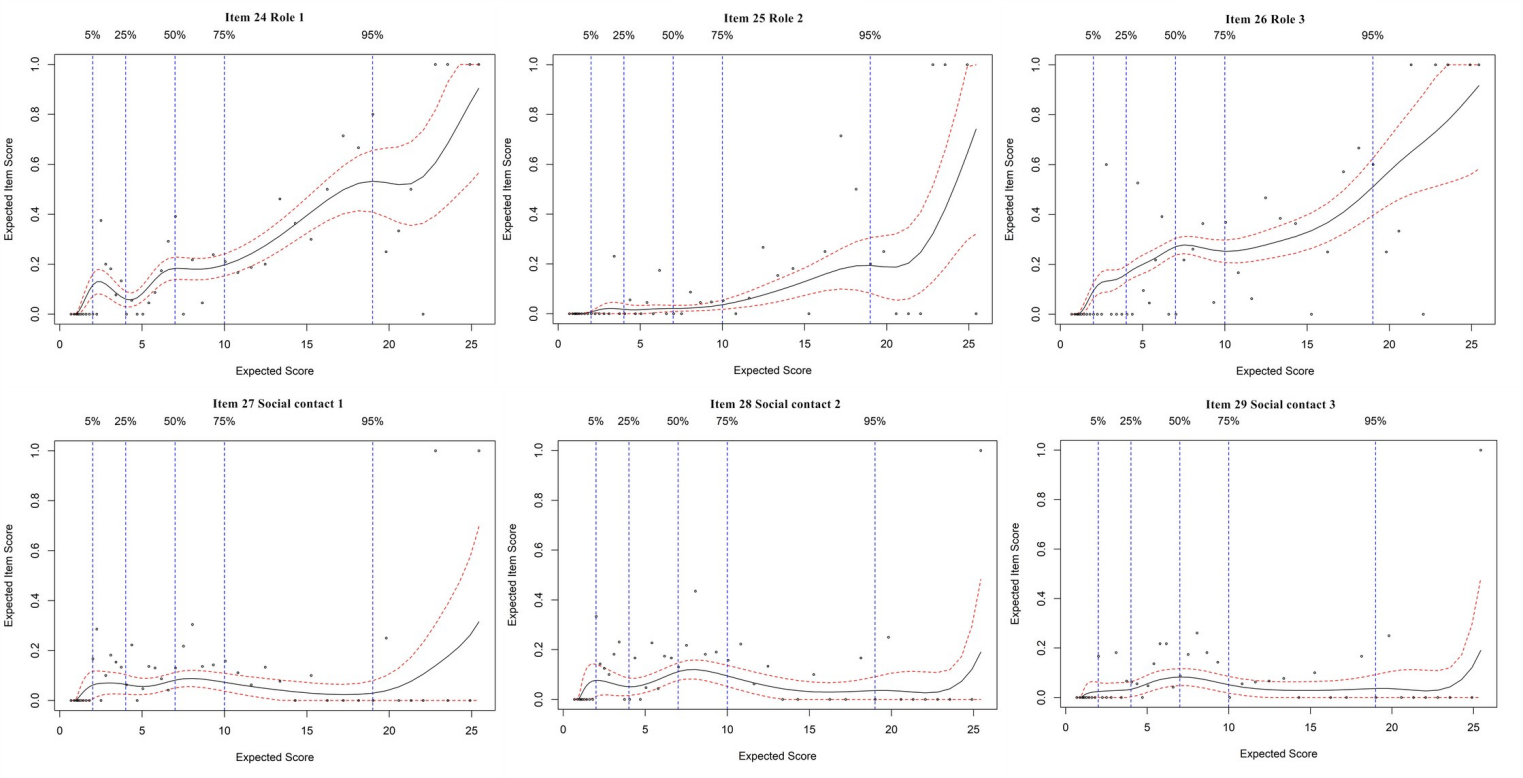
Expected item score curves for social domain.

An EIS curve analysis was employed for each item of the FI-35. The EIS curves of the 35 items are presented in Figs 1–4. Since most of the EIS curves increase across the entire frailty level, most of the items differentiate relatively well across the entire frailty range. From a detailed study of the EIS curves represented by the 35 plots, our results show two findings: a. some items within a domain contribute to a similar extent; b. some items in the same subdomain are different. The different items differentially contribute to the general level of frailty.

**Fig 5:**
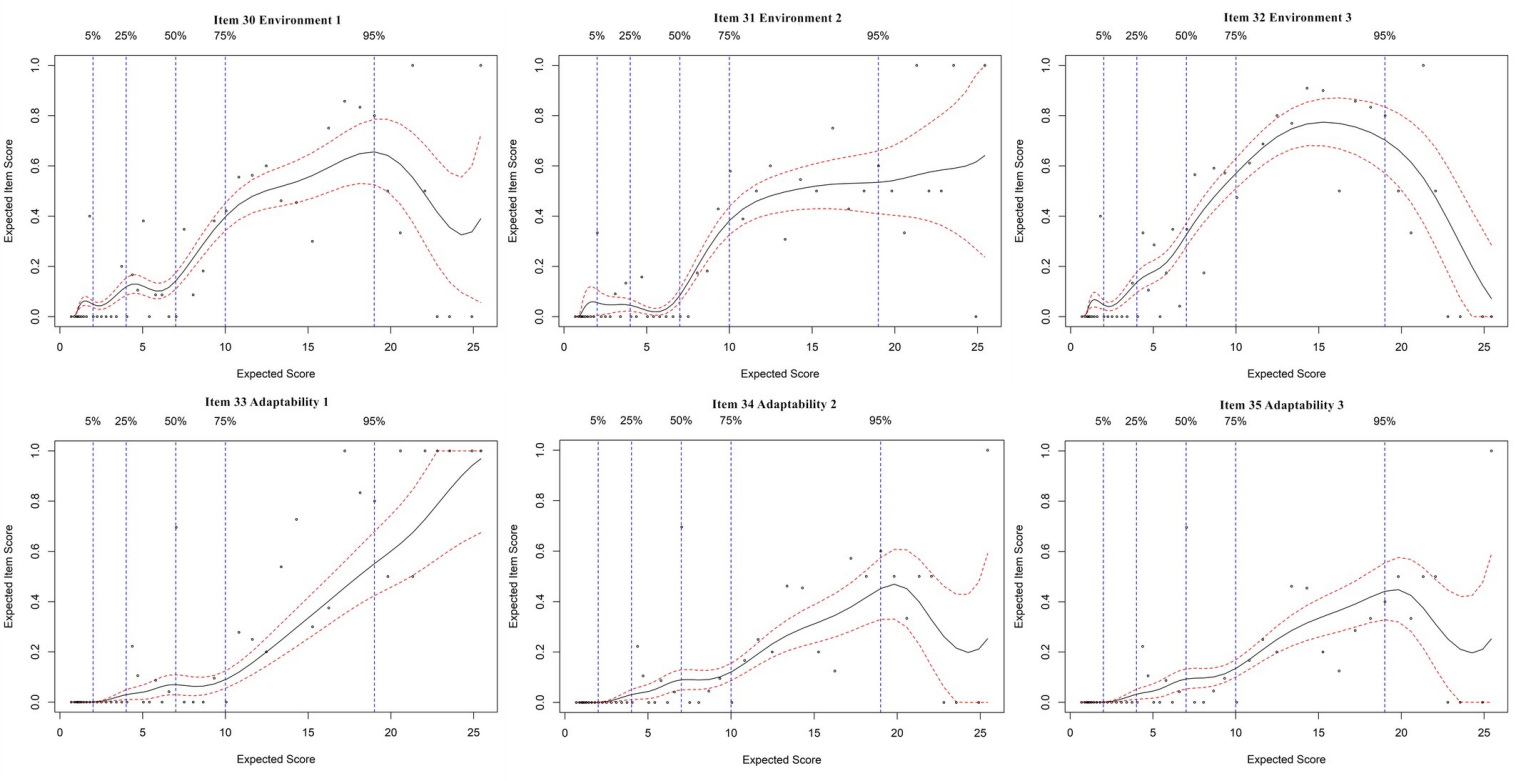
Expected item score curves for environment domain.

The items in the nutrition, muscle strength, vigor, emotion, and role subdomains all showed the same trend in their EIS curves, which indicated that all of the items in these subdomains express the same characteristic regarding frailty level. Looking into the curves in more detail, the first, second and third items in the exercise subdomain showed a relatively steep increase in the curve, indicating that as the level of frailty increases, older adults tended to walk more slowly and experience more difficulty going upstairs (show in Fig 2). However, the fourth item in the exercise subdomain showed a relatively horizontal curve; this item (engaging in some energy-consuming activities) did not differentiate well between moderately and highly frail persons. Also in Fig 2, the subdomain of sleep quality, the second and third items showed the same trend in the EIS curves with a sharp increase between 2 and 8; that is, the items “your sleep is more interrupted than before” and “you need medication to help you sleep” differentiated very well between non-frail and moderately frail individuals. However, the first item of the sleep quality subdomain sharply increased at a score of 20 and above, indicating that “getting less sleep” differentiated highly frail individuals. Similarly, in Fig 3, three items in the cognition subdomain (items 20, 21, and 22) showed relatively steep increases in the curves across each level of frailty; however, the fourth item, “it is harder to focus on one thing than before”, showed a sharp increase between 0 and 4, followed by a relatively horizontal curve between moderate and high frailty, indicating that this item differentiated well in the very early stages of becoming frail.

Furthermore, in Fig 5, the first item in the environment subdomain, “you are worse at adapting to temperature changes”, showed a sharp increase in the EIS curve across the scores of 6–17; the second item, “you are more sensitive to the environment”, showed a sharp increase in the EIS curve across the scores of 6–14; and the third item, “your living condition is more convenient than before you live”, showed a sharp increase in the EIS curve across the scores of 4–15. These data indicated that all the items in the environment subdomain typically increased for older people with low frailty scores; that is, they differentiated very well between non-frail and moderately frail individuals. However, the first and third items in the environmental subdomain showed a sharp decrease in the curve at scores of 18 and above (in Fig 5), indicating that “worse at adapting to temperature changes” and “more convenient living condition” were not significant in highly frail individuals. In Fig 5, the subdomain of adaptability, the second and third items showed the same trend in the EIS curves and discriminated well between non-frail and moderately frail individuals, but the first item showed a relatively steep increasing curve across each level of frailty.

## Discussion

This is the first detailed analysis of the contribution of individual items to frailty. The detailed item score curves revealed that almost all items contributed in a monotonically increasing manner to the generic concept of frailty. From a detailed study of the EIS curves represented by 35 plots, our results show three findings:

- The EISs increased monotonically for the vast majority of the items, with the exception of the social contact items;
- Nearly all items within a subdomain behaved similarly;
- Some items differentiate very well between sub-ranges of frailty.

It was found that within the physical domain, the exercise and muscle strength subdomain items differentiated over the whole frailty range. This finding is consistent with previous studies showing that older people’s gait speed and grip strength can be used to measure frailty [20,21]. A short physical performance battery was used by Kim [20] to examine the relationship of the walking speed test with different distances or paces to health-related factors in older adults. Thus, we can take the decreases in gait speed and grip strength into account, but these are specifically useful at low levels of frailty to identify when early interventions will decrease frailty progression. Other items, for instance, the items in sleep quality subdomain, differentiate very well within a sub-range of frailty: item 14 "you’re getting less sleep" differentiates very well between moderate and highly frailty, item 15 “your sleep is more interrupted than before” and item 16 “you need medication to help you sleep” show a relatively steep increasing curve between non-frail and moderately frail regions. A previous study revealed that poor sleep quality was associated with frailty in an older population [22]. Appropriate interventions have significant effects on regulating mood in older adults, resulting in relaxed and happy states of mind and improved confidence in life [23]. In our research, lighter sleep occurs in the early stages of frailty; thus, early screening and appropriate intervention for sleep disorders in clinical practice are necessary to improve fatigue, slow down the development of frailty and improve the quality of life of older people.

Within the psychological domain, the first three items in the cognition subdomain differentiated across the whole frailty range. Several previous studies have shown the relationship between frailty and cognition. Prefrailty is a risk factor for cognitive dysfunction, and cognition tends to decline as frailty increases [24–27]. A meta-analysis from Bu [28] summarized that cognitive frailty was found to be a predictor of dementia in older adults living in communities. In our work, the abilities of memory, expression and solving problems were representative of cognitive impairment. Thus, maintaining cognitive functional abilities is important, and we suggest improving these abilities using memory, expression and solving problems as strategies to reduce disease-related cognitive impairment and improve frailty in older adults. The fourth item in the cognition subdomain, “it is harder to focus on one thing than before”, showed a relatively steep increase in the curve between the low and moderately frail regions. That is, inattention is more likely to occur in the early stages of frailty. Thus, cognitive decline is a factor in the early stages of frailty and should be attended to in all older people to prevent and ameliorate frailty. Furthermore, the items in the emotion subdomain behaved similarly and differentiated very well high levels of frailty. Mulasso [29] suggested that negative emotions were correlated with and aggravated frailty. In our study, the items in the emotion subdomain showed a relatively steep increase in the curve in the highly frail region. Therefore, it is necessary to evaluate the level of negative emotion in prefrailty and avoid the progressive deterioration to high frailty.

In the social domain (in Fig 4), the items in the role subdomain increased across the entire range of frailty levels. Psychological disorders in older adults are most likely to prevent them from adapting to social and family changes after retirement and leave them feeling upset, with nothing to do and perhaps depressed [30]. From the perspective of the family and society, it is important to encourage them to independent participate in activities. The progression of frailty among older people might be slowed through the engagement in appropriate activities. However, the EIS curves in the social contact subdomain did not discriminate over the whole frailty range; that is, the level of frailty did not increase with decreased social contact, which was different from previous research [31,32]. This may be because the participants were recruited from communities in Tianjin, and most of them were in middle/high income range with hobbies and health activities in society.

Regarding the environment domain, all the items in the environment subdomain differentiated very well between non-frail and moderately frail individuals; that is, older adults at low frailty levels were sensitive to the environment. Thus, it would be beneficial to maintain an appropriate environment. However, it is interesting that the third item “your living condition is more convenient than before you live” showed a decreasing trend in the EIS curves in highly frail individuals. It may be that this represented a decrease in activities among highly frail adults, and a decrease that was a requirement based on their surroundings. Therefore, the environment was not significant in highly frail adults.

Most studies based on total frailty scores or in different domains of frailty have developed interventions [33,34] while ignoring the significance of individual items. What’s more, sever researches also ignore the comparison with other frailty indexes and the individual. It is important that the multidimensionality as well as the different patterns of frailty are recognized in the clinical setting to improve care for older persons. This study determined the contribution of individual items to frailty by showing that their contribution to the total FI-35 score can differ largely depending upon the level of frailty.

## Conclusions

Although nearly all the items monotonically increased with frailty levels, there were large differences between items in their ability to differentiate between persons being either weakly, moderately or highly frail. The quality of interventions of care and counseling can be improved by taking into account the specific ability of items to differentiate within specific frailty levels. Thus, different intervention measures should be adopted for older people with different levels of frailty.

## Supporting information

S1 Data (SAV)
